# The Kaleidoscope of Microglial Phenotypes

**DOI:** 10.3389/fimmu.2018.01753

**Published:** 2018-07-31

**Authors:** Marissa L. Dubbelaar, Laura Kracht, Bart J. L. Eggen, Erik W. G. M. Boddeke

**Affiliations:** Department of Neuroscience, Section Medical Physiology, University of Groningen, University Medical Center Groningen, Groningen, Netherlands

**Keywords:** microglia, activation, phenotype, transcriptome, neurodegenerative diseases, core gene signature

## Abstract

Gene expression analyses of microglia, the tissue-resident macrophages of the central nervous system (CNS), led to the identification of homeostatic as well as neurological disease-specific gene signatures of microglial phenotypes. Upon alterations in the neural microenvironment, either caused by local insults from within the CNS (during neurodegenerative diseases) or by macroenvironmental incidents, such as social stress, microglia can switch phenotypes—generally referred to as “microglial activation.” The interplay between the microenvironment and its influence on microglial phenotypes, regulated by (epi)genetic mechanisms, can be imagined as the different colorful crystal formations (microglial phenotypes) that change upon rotation (microenvironmental changes) of a kaleidoscope. In this review, we will discuss microglial phenotypes in relation to neurodevelopment, homeostasis, *in vitro* conditions, aging, and neurodegenerative diseases based on transcriptome studies. By overlaying these disease-specific microglial signatures, recent publications have identified a specific set of genes that is differentially expressed in all investigated diseases, called a microglial core gene signature with multiple diseases. We will conclude this review with a discussion about the complexity of this microglial core gene signature associated with multiple diseases.

## Introduction

Macrophages are innate immune cells that reside in all organs of the body. They have versatile functions that are tailored to the organ of residence (1). Genome-wide studies showed that microenvironment-specific signals establish tissue-specific properties of macrophages *via* epigenetic mechanisms (2, 3).

Transcriptomic analyses are an effective way to determine gene expression patterns that serve as a proxy for different cellular states under different conditions. The last decade, numerous transcriptome studies of (micro)glia have been published and provide much insight in glia biology (4, 5).

Gene expression profiling of purified microglia has confirmed that they are central nervous system (CNS)-resident macrophages that express many genes typical for the myeloid lineage, including receptors for pathogen-associated molecular patterns and damage-associated molecular patterns, genes involved in phagocytosis and antigen presentation. This makes distinction between microglia and macrophages, particularly under neuropathological circumstances, very difficult (6). A common approach to separate microglia from other cells of the periphery and CNS is the preparation of a single-cell suspension followed by fluorescence-activated cell sorting (FACS) based on the membrane expression of CD11b^high^ and CD45^low/int^ in mice and human (7). In mice, Ly6C/Ccr2 and Mrc1 are specifically expressed by monocytes (8) and CNS interface macrophages (9), respectively and can be additionally used to distinguish between these cells and microglia. In humans, although not yet widely applied, CCR2 and CD14 are used to discriminate between microglia and monocytes (10).

In recent years, RNA expression profiling of microglia has received much attention (see the glia open access database) (11). This review will focus on microglial phenotypes in the CNS related to manifold processes associated with brain development, physiology, and pathology.

## Microglia Origin and Homeostasis

### Microglia Ontogeny

As already proposed by Río-Hortega in 1919 (12), sophisticated *in vivo* lineage tracing studies confirmed the mesodermal origin of microglia during embryogenesis (13–15). This is different from other CNS cells that arise from the neuro-ectoderm (16). Even within the mesoderm-originating myeloid cell compartment, microglia have a distinct ontogeny. In mice, tissue-resident macrophages emerge from two waves of erythromyeloid progenitor (EMP) production (primitive and transient definitive hematopoiesis) in the extra-embryonic yolk sac (YS) prior to the establishment of definitive hematopoiesis in the fetal liver and later in adult bone marrow (14, 17). Microglia originate from the primitive hematopoietic wave of early EMP’s (primitive macrophages) at embryonic day 7.5 (E7.5) in the YS—a process dependent on the transcription factors (TFs) *Spf1* (*Pu.1*) and *Irf8* (14, 15).

These primitive macrophages spread *via* the bloodstream to the developing organs, including the neurepithelium, which gets colonized by primitive macrophages (microglia) as early as E9.5 (13). By contrast, other tissue-resident macrophages mainly develop from the transient definitive hematopoietic wave of late EMP’s arising at E8.5 in the YS. These late EMP’s subsequently colonize the fetal liver from E10 onward and mature into tissue macrophages *via* a monocytic intermediate (14, 17). Currently, it is not yet resolved why these differences in microglia and macrophage ontogenies exist.

Although human microglia ontogeny is not yet studied in such detail, immunostaining of the human encephalon indicates the presence of IBA1 positive microglia at gestational week 5.5, that enter the brain *via* the ventricles. Microglia proliferate and develop toward their typical ramified morphology from that time point onward (18). However, the ontogeny of human microglia remains to be defined in detail.

### Microglia Development Occurs in Four Consecutive Stages

A recent study combining transcriptome with epigenome analysis identified genes and chromatin modulators that regulate different stages of microglia development in mice (19). Microglial gene expression clusters are identified that are specific for four sequential developmental phases: YS (E10–12.5), early microglia (E10.5–14), pre-microglia [E14—postnatal day 9 (P9)], and adult (P28 onward). Early, pre- and adult microglia are marked by genes related to cell cycle and proliferation (*Dab2, Mcm5*, and *Lyz2*), synapse pruning (*Crybb1, Csf1*, and *Cxcr2*), and immune surveillance (*Mafb, Cd14*, and *Mef2a*), respectively. These developmental stage-specific gene ontology (GO) terms (unifying terms annotating a global function to genes) match with typical microglia functions, including the involvement in neuronal network refinement (synapse pruning) (20, 21) and maintenance of adult brain homeostasis (22). Extensive parallel single-cell sequencing of microglia identified a high degree of homogeneity of microglia populations at specific developmental stages. Concordantly, the expression level of developmental stage-specific genes correlate to the accessibility of corresponding enhancers identified by dimethylation of lysine 4 on histone 3 (H3K4me2)-enriched regions distal from the transcription start sites of a gene. Whereas YS and embryonic microglia cluster more closely together at the transcriptional level, embryonic and pre-microglia cluster more closely together at the epigenetic level. These results indicate that the microenvironment is driving gene expression through modulation of the epigenetic landscape into a permissive state for the expression of gene patterns belonging to specific developmental phases. This suggestion is corroborated by the fact that environmental perturbations, such as in germ free (GF) mice and maternal immune activation, led to abnormal microgliosis. Mice that are subjected to maternal immune activation display a shift from the pre-microglia stage toward a more advanced developmental stage, due to a decreased expression level of inflammatory and defense related genes. It is hypothesized that the disruption of microglia development disturbs physiological microglial functions (19).

In addition, specific potential TF binding motifs are identified in promotor regions of genes specifically expressed at different microglial developmental phases. Clearly, the previously identified TFs *Pu.1* and *Irf8* are essential for microglia development (15) and are highly upregulated throughout microglia development (19). These results corroborate the finding that *Pu.1* is essential for the gene regulation of several functions including myeloid cell differentiation, chemotaxis, and phagocytosis (23–25). In line with the findings of *Irf8* in the study of Kierdorf and coworkers (15), *Irf8* is also important in myeloid cell lineage differentiation and survival during early hematopoiesis in zebrafish (26).

The TF *Mafb* is enriched in adult microglia and the identification of immune and viral GO terms, enriched in the pre- and adult microglia signature of *Mafb*-deficient microglia, suggests that *Mafb* is crucial for the regulation of brain homeostasis.

In a similar approach, consistent findings according to microglial developmental stages and stage-specific functions were recently identified (27). Interestingly, it was shown that microglia progenitors in the YS and at E10.5 already express a part of the homeostatic microglial signature genes (see The Homeostatic Microglial Gene Signature in Mice and Men), which then expand with increasing developmental stage. Based on genes expressed at all developmental stages, a murine microglia development signature containing 568 genes was identified and compared to gene expression data of FACS-purified microglia from human fetuses ranging from 14 to 24 weeks of estimated gestational age. This analysis identified 387 overlapping genes, involved in functions as immune response and phagocytosis. In addition, it was shown that microglia derived from E16.5 mice are developmentally corresponding to microglia derived from mid-trimester human fetuses (14–24 weeks of pregnancy). Although these human microglia already express genes belonging to the homeostatic microglial gene signature, it should be of note that human fetal microglia do not (yet) seem to be sexually dimorphic (see Microglial Sexual Dimorphism) (27).

Of importance, it seems that deviations in the microglial developmental transcriptome are linked to the development of neurological diseases, such as autism and Alzheimer’s disease (AD) in adulthood (28).

In conclusion, microglial development occurs in a complex and fine-tuned sequence of processes regulated by environmental signals and is associated with specific gene expression programs.

### The Homeostatic Microglial Gene Signature in Mice and Men

Over the last years, the transcriptome of homeostatic murine microglia was identified (2, 3, 29–33). Under homeostatic conditions, this transcriptome contains genes specifically expressed by microglia in comparison to other CNS cells and myeloid cells, hereafter referred to as homeostatic microglial signature genes. These genes are now widely used by other researchers to identify and study microglia, also under disease conditions (see Box 1).

Box 1 **Environmental influence on the microglial homeostatic gene signature.**

Evidence is accumulating that genes belonging to the homeostatic microglial gene signature are downregulated during neurological diseases. It was shown that microglia uniformly downregulate their homeostatic signature genes, such as *Sall1, Pu.1, Tmem119, Cx3cr1*, and *P2ry12/13* and upregulate risk genes for AD, such as *Apoe* and *Trem2*, in mouse models for aging, AD (5XFAD and APP-PS1), and amyotrophic lateral sclerosis (ALS) (SOD1) (34–36). Interestingly, homeostatic microglial signature genes seem to be differentially expressed at different disease stadia in EAE, a mouse model for multiple sclerosis (MS). A downregulation of homeostatic microglial signature genes is observed in acute and chronic EAE, whereas during the recovery phase of EAE, gene levels are restored to those of homeostatic microglia (34, 37). Concordantly, a loss of homeostatic microglial signature genes is identified in human MS brain tissue (38). Furthermore, homeostatic microglial signature genes seem to be at least partially downregulated during aging (33, 39, 40) and are differentially expressed in male and female murine microglia (27).

In addition, it was shown that microglia upregulate CD45 expression under different disease conditions (8, 41). Furthermore, monocytes downregulate Ly6C and Ccr2 during their differentiation into macrophages after infiltrating brain tissue (6, 8), resulting in issues regarding the distinction of microglia and peripheral monocyte/macrophages in disease conditions.

Concordantly, the use of specific markers to identify microglia under specific disease conditions is still controversial.

#### The Homeostatic Gene Signature of Murine Microglia

The first gene expression profile of murine microglia was obtained in 2012 in a microarray study (32). Based on these expression data, that included a common macrophage signature, several gene clusters were identified. However, distinct gene expression signatures among four different macrophage populations were identified. 64 genes, including *SiglecH* and *Cx3cr1* were shown to be more abundantly expressed in microglia when compared to other investigated macrophage types. Chiu et al. identified 29 genes that are highly specific for microglia, including *Olfml3, Tmem119, and SiglecH* (31).

Direct RNA sequencing revealed the microglial sensome, consisting of 100 cell surface receptors and proteins specific for the sensing of microenvironmental factors, including pattern recognition-, chemokine-, Fc-, purinergic-, cytokine-, extracellular matrix-, and cell–cell interaction receptors (33). Approximately half of these genes seem to be regulated by Tyrobp (Dap12), a protein tyrosine kinase binding protein and ligand for Trem2, both belonging to the homeostatic microglial signature genes (33). The Trem2–Dap12 signaling pathway seems to be involved in (1) the suppression of toll-like receptor (TLR)-induced inflammation, (2) mediating phagocytosis, and (3) reduction of cell death and enhancement of myeloid cell proliferation (42). Analogous to other studies, Hickman and coworkers identified several genes that are shared by microglia and other tissue macrophages, but also macrophage subtype-specific expression of gene sets. The top 25% uniquely expressed genes in microglia contain many of the sensome-including genes (33).

In 2014, two studies were published that extensively investigated microglia and other tissue-resident macrophages at transcriptome level (2, 3). These studies also address the epigenetic differences between different macrophage subsets, observing a positive correlation with the transcriptome. When comparing large peritoneal macrophages (LPM), small peritoneal macrophages (SPM) and microglia at the transcriptional level, both macrophages and microglia are depended on *Pu.1*. However, the co-enrichment of different motifs was revealed, LPM and SPM are thus shown to be depended on retinoic acid (RA) receptors (RAR α/β) whereas motifs as SMAD, consistent with the TGFβ signaling in the brain is shown to be unique for microglia (2). Analysis of seven different tissue-resident macrophage populations identified 3.348 differentially expressed genes. K-means clustering of these genes led to the discovery that the microglial cluster (consisting of 641 genes that are higher expressed in microglia) is different from other tissue macrophages, where *Sall1* is found to be most highly expressed in microglia (3).

A Tgf-β-dependent homeostatic microglial gene signature consisting of 152 unique microglial genes, *P2ry12, Tmem119, Fcrls*, and three microRNAs (miRNAs) is identified by comparing the microglial transcription profile to that of other CNS cells and monocytes. The validity of these genes being uniquely expressed in microglia is confirmed by mass spectrometry, since many of these genes are also detected in the enriched fraction of microglial proteins. It is shown that Tgf-β is a crucial factor for the establishment of the microglial homeostatic gene signature, since mice that endogenously lack Tgf-β in CNS tissue show a remarkable reduction in microglial numbers and the remaining microglia show significantly reduced expression of these homeostatic microglial signature genes (30).

A transcriptome profile of isolated microglia that closely approximates their *in vivo* status was published by Bennett and coworkers, using a relatively non-invasive method to purify microglia (29). Inflammation-associated genes (*Il1b, Nfkb2*, and *Tnf*) are significantly lower expressed in this dataset compared to others (2, 31, 32), indicating that *in vitro* procedures influence the homeostatic microglial gene signature (see The Transcriptome of *In Vitro* and *Ex Vivo* Microglia Is Different). Tmem119 was studied in detail and was identified to be a specific and, at least at protein level, robust microglial marker in mice and human, also under inflammatory/disease conditions. In addition, potential novel microglial functions associated with vascular development (*Pdgfb*), oligodendrocyte development (*Pdgfa*), and synapse formation (*Sparc*) are identified and microglial involvement in different neurological diseases (*Comt, Hprt*, and *Trem2*) are confirmed by the enrichment of the indicated genes in microglia (29).

The common denominator of at least these seven studies is the identification of the homeostatic microglial signature genes, including *Sall1, Hexb, Fcrls, Gpr43, Cx3cr1, Tmem119, Trem2, P2ry12, Mertk, Pros1*, and *SiglecH*, that are uniquely/higher expressed in microglia and not or only at low levels in other brain cells or myeloid cell types, including tissue-resident macrophage subsets and monocytes.

#### The Homeostatic Gene Signature of Human Microglia

In parallel to the identification of homeostatic microglial signature genes in mice, two studies identified homeostatic gene signatures of human microglia. Gosselin and coworkers investigated the transcriptomes of microglia purified from healthy-appearing brain tissue obtained during neurosurgery of 19 young patients (0–17 years) with epilepsy, tumors, or acute stroke. The top 30 highly expressed genes in that dataset are related to functions like microglial ramification and motility (*P2RY12* and *CX3CR1*), synaptic remodeling (*C3* and *C1QA-C*), and immune response (*HLA-DRA* and *HLA-B*). The comparison of microglia-specific and whole cortex gene expression profiles identified 881 homeostatic human microglial signature genes, including *CX3CR1, P2RY12*, and several complement factors as *C3, C1QA, C1QB*, and *C1QC*. In addition, these human microglial homeostatic signature genes significantly overlap with transcriptomic datasets related to different neurological diseases, including AD and Parkinson’s disease (PD), in which many of the human homeostatic microglial signature genes are differentially expressed, indicating an important role of microglia in the pathophysiology of these diseases (43).

Another study identified the homeostatic human microglial gene signature from a population of 39 adult (34–102 years) postmortem donors in The Netherlands and Brazil. This homeostatic microglial gene signature is characterized by 1,297 genes that are significantly differentially expressed in purified microglia when compared to whole parietal cortex cell lysates. GO analysis indicated that these genes are related to the innate immune system, including functions as pathogen and self-recognition, inflammasome, cell adhesion and motility (C3XCR1), immune signaling and modulation (*P2RY12, Q1QA-C*, and *HLA-DR*). In addition, risk genes for neurodegenerative diseases, such as *APOE* and *TREM2* are enriched in purified adult human microglia (39). In addition, the two TFs *PU.1* (*SPI.1*) and *IRF8*, which are also crucial during murine microglia ontogeny and development (19) are highly expressed in both datasets (39, 43).

Thus, together these two studies identified the homeostatic human microglial gene signature, that shares many genes with the murine homeostatic gene signature, but also seem to possess human-specific properties.

The homeostatic microglial gene signature is conserved across species (39, 43). Comparison of the two homeostatic human microglial gene signatures to several homeostatic murine microglial gene signatures reveals an overlap of more than 50%, depending on the specific datasets that were compared (39, 43). The genes *APOC1, MPZL1, SORL1, CD58, ERAP2, GNLY*, and *S100A12*, most closely related to the innate immune system, are specifically found to be expressed in human microglia and not, or only to a very low extent in murine microglia. Concordant with the high overlap between murine and human transcriptomes are the identified similar epigenetic landscapes, i.e., identified microglial-specific regulatory regions, in murine and human microglia (43). Concluding, research of recent years has identified the homeostatic murine and human microglial gene signatures, which enables better identification and investigation of microglia in murine and human tissue.

### Microglial Sexual Dimorphism

Sex-specific transcriptomic signatures are found when comparing adult male and female mice. A higher gene expression level of inflammatory response genes, such as *Ccl2, Tnf, Irf1, Cxcl10, and Il1b* is found in female mice, indicating a more immune-activated state of female microglia. In addition, homeostatic microglial signature genes are differentially expressed in male and female mouse microglia. Interestingly, it is demonstrated that environmental alterations during embryogenesis, like the absence of the maternal microbiome (GF mice), has different effects on male and female microglial transcriptomes at the identified developmental stages (see Microglia Development occurs in Four Consecutive Stages). Whereas the transcriptome of microglia from GF offspring does not seem to be overtly altered at E14.5 when compared to control microglia (under specific pathogen-free conditions), it is affected at E18.5 especially in males and in adults especially in females, characterized by 1,216 and 433 differentially expressed genes, respectively. From those 1,216 differentially expressed genes in GF E18.5 males, the majority is downregulated and involved in functions such as translation, endocytosis, and metabolism. Regarding the 433 differentially expressed genes in GF adult female microglia, approximately half of these genes are downregulated and involved in the inflammatory response, whereas the upregulated genes are associated with the regulation of transcription. In addition to transcriptomic changes, the pattern of microglial colonization into the neocortex also occurs in a sex-dependent manner in offspring from GF dams. Whereas male offspring of GF dams show an increased microglial density prenatally (E18.5), female offspring of GF dams show an increased microglial density postnatally (P20) (27).

Concordant with the findings by Thion and coworkers a developmentally more mature state, marked by upregulation of genes involved in immune processes, is identified in female microglia compared to male microglia at P60. This result is based on the microglial developmental index (MDI) that is calculated by the ratio of the average expression of globally upregulated genes divided by the average expression of globally downregulated genes in a developmental time course from E18 to P90 in male and female mice (28). Upon lipopolysaccharide (LPS) treatment, the MDI of male microglia increases, indicating maturation of male microglia in response to LPS. These sex-dependent baseline and LPS-induced changes in the transcriptome are accompanied by sex-specific microglial morphologies in the adult hippocampus of mice. When compared to the morphology of baseline female microglia, baseline male microglial morphology seems to be more complex. It is marked morphologically by an increased process volume and area and an increased number of branches and intersections, albeit female and male differences are only statistically significant for the parameter process volume. Upon LPS stimulation, baseline morphological characteristics of male microglia get significantly reduced, whereas those of female microglia do not change much (28).

Concluding, murine microglia seem to respond to environmental insults in a sex-dependent manner, which was not yet manifested in human microglia (27).

### Microglia Possess Brain Region-Specific Transcription Profiles

Insight in regional heterogeneity of microglial phenotypes can provide necessary information on regional specific microglial functions. RNA sequencing of bulk samples containing large numbers of microglia from the whole brain might mask specific regional heterogeneity. Whereas microglia are important for general functions including scanning the microenvironment, phagocytosis, and neuronal support (22), specific, additional regional microglial functions could be envisioned.

Several mouse brain regions were compared, exploring the hypothesis that the microenvironment could shape microglial functions (40). Regional transcriptional heterogeneity is observed when microglia from the mouse cerebral cortex, hippocampus, cerebellum, and striatum are compared. Three transcriptomic clusters are identified, specific for cerebral cortex/striatum, hippocampus, and cerebellum. Annotation of associated biological processes revealed that the hippocampal microglial gene cluster is involved in energy production and regulation, whereas the cerebellar and cortical clusters are associated with genes involved in immune response and regulation. Concordantly, TF binding motif analysis found TFs regulating the expression of bioenergetic genes and immune and inflammatory genes to be over-represented in the hippocampal- and the cerebellar cluster, respectively. Interestingly, there seems to be a difference in the immune-activation state of microglia belonging to the cortical and the cerebellar cluster. Cortical microglia show an increased expression of genes coding for inhibitory immunoreceptors, including *Trem2* and *SiglecH*, whereas cerebellar microglia show an upregulation of genes coding for activating immunoreceptors, indicating a more immune-activated microglial phenotype, different from the LPS or IL-43-induced microglial phenotypes. Notably, approximately one-third of the microglial sensome genes (belonging to the homeostatic microglial gene signature) are differentially expressed in microglia derived from different brain regions. Concluding, although microglia from different brain regions share the expression of specific genes, they also express region-specific gene sets indicating region-specific microglial functions (40).

De Biase and coworkers reported different microglial phenotypes when comparing regions in the basal ganglia. The transcriptome of ventral tegmental area (VTA) microglia appears to be most distinct when compared to microglia in the nucleus accumbens and substantia nigra pars compacta (SNc). Differentially expressed genes in microglia of the VTA are involved in metabolic processes such as mitochondrial function, glycolysis, gluconeogenesis, and oxidative phosphorylation. Microglia in the VTA and SNc show limited surveillance and contribution in homeostasis, based on observations made in cell density, branching, and lysosome content. Based on the overlapping microglial genes in the different regions, classical microglial cell functions are preserved among the different regions. However, microglia in different regions also exhibit regional adaptation (44), a finding consistent with that of Grabert and coworkers.

In another study, microglia were compared with non-parenchymal CNS macrophages in the subdural meninges, perivascular spaces, and the choroid plexus on single-cell transcriptome level. Gene expression profiles of microglia and the three investigated CNS interface macrophage populations display high similarity in contrast to peripheral monocytes. When compared to the monocytic transcriptome, microglia and non-parenchymal macrophages share 443 differentially expressed genes, such as abundant expression of the myeloid markers Cx3cr1, Csfr1, and *Aif* (*Iba1*). The high overlap of transcriptomes between these brain-associated macrophages might be based on their similar ontogeny and kinetics, since perivascular and meningeal macrophages, analogous to microglia, also arise during primitive hematopoiesis in the YS and are long-lived cells that do not get replenished by peripheral monocytes. Besides this commonly expressed gene set, microglia and non-parenchymal macrophages also express unique separate gene sets. Microglia show differential expression of 2,328 unique genes that are unaltered in expression in non-parenchymal macrophages after comparison to the monocytic transcriptome. As an example, *P2ry12* and *Mrc1* are enriched in microglia and perivascular macrophages, respectively, and thus are used to distinguish these brain-associated macrophage populations (9).

Although not studied such extensively, it seems that human microglia also show brain region-specific gene expression profiles (45).

Concluding, the CNS is populated by different macrophage cell types, and even microglia in the parenchyma can be subdivided into different phenotypes based on their gene expression profiles, which might be associated with specific functions.

### The Lifetime of Microglia

Microglia, as well as other tissue-resident macrophages (46, 47), are stable, self-renewing cell populations over the entire lifespan of an animal. The self-renewing capacity of microglia has been shown in an experiment where microglia were ablated using the Cx3Cr1*^CreER^*:iDTR system. Within 5 days the 20% remaining microglia completely repopulated the CNS (48). This process was independent of infiltration of peripheral monocytes but was dependent on microglial interleukin-1 signaling. In a similar experiment, where treatment with a macrophage Csf1r inhibitor caused ablation of 99% of the resistant microglia, a full repopulation of microglia *via* nestin-positive progenitors within 1 week after treatment was observed (49). While it is well accepted that, at least under physiological conditions, microglia are not replenished by peripheral macrophages, the lifetime of microglia is still a matter of debate. Askew and coworkers (50) reported that microglia are rather fast proliferating cells with a turnover rate of approximately 3 months. By contrast, Füger and coworkers and Tay and coworkers propose cortical microglia to be long-lived cells with turnover rates between 15 and 41 months, respectively (51, 52). Turnover rates of microglia seem to vary between brain regions (50–52).

Although studying the lifetime of microglia in humans comes along with experimental limitations, estimations of human microglia turnover rates was made. It was estimated that the human microglia population might renew several 100 times within the average human lifetime of 80 years (50). By contrast, a relatively slow mean microglial turnover rate of approximately 28% per year and an average microglial age of 4.2 years is calculated using thymidine analog IdU (5-iodo-2′-deoxyuridine) labeling in brains of cancer patients and retrospective atmospheric ^14^C measurements in the DNA of the same and healthy tissue postmortem (53). Clearly, the different microglial turnover rates that have been reported may be caused by the use of different methodologies and these findings need to be reconciled in the future.

Under disease conditions, it has been observed that microglia can transiently be replenished by monocyte-derived macrophages from the periphery, especially when the blood–brain barrier is disrupted (54, 55). In addition, turnover rates of microglia are increased under neurodegenerative conditions such as in the APP/PS1 mouse model for AD [containing AD risk mutations in the genes encoding for the amyloid beta precursor protein (*App*) and presenilin (*Psen1/Psen2*)] (51), unilateral facial nerve axotomy (FNX) in mice (52), and nitroreductase (NTR)-induced neurodegeneration in zebrafish larvae (55). Interestingly, during the resolution phase of neuroinflammation and -degeneration there seems to be a self-regulating mechanism returning microglial numbers in the CNS to physiological conditions. Intravital and electron microscopy of zebrafish larvae brains 8 days after NTR-induced neurodegeneration has shown that phagocytes (microglia and peripheral macrophages) either leave the CNS tissue with unknown destination or undergo apoptosis and are phagocytosed by viable microglia leading to a physiological microglia density in the forebrain numbers resembling those of healthy zebrafish (55).

In addition, microglial migration into regions distal to the neurodegenerative center as well as microglial apoptosis may contribute to the re-establishment of a homeostatic-like microglia density in the mouse brain during the resolution phase of unilateral FNX-induced neurodegeneration. The increased phagocytic activity of microglia identified by confocal microscopy as well as RNA sequencing during later phases of neurodegenerative resolution, led to the hypothesis that also in mammalian species microglia self-regulate their density by phagocytosing excessive microglia that have undergone apoptosis. After the resolution of neurodegeneration, a mixture of microglia that already existed and newly proliferated microglia is preserved (52).

Currently, it is not yet known whether a replicated cell is biologically younger or if it inherits (epi)genetic marks from the mother cell (51). It was shown that LPS treatment during embryonic development results in a dampened immune response (LPS tolerance) in the same mice when they are young adults (56), indicating that deviations in early microglial development have long-lasting effects on the microglial phenotype during aging and associated diseases.

## The Effect of *In Vitro* Conditions on the Homeostatic Microglial Gene Signature

### Human iPSC (hiPSC)-Derived Microglia-Like Cells

Restricted numbers of human microglia can be obtained by purification from human postmortem and surgically resected brain tissue. Unfortunately, limited access to viable human brain tissue causes a challenge to study human microglia and their respective roles in neurobiological diseases. One possibility to study human microglia on a relatively large-scale is the recent development that microglia-like cells can be generated from induced pluripotent stem cells (iPSCs). Thus, various studies describe protocols for the differentiation of hiPSCs into microglia-like cells. These differentiation protocols recapitulate microglial embryonic development and, in some cases, physiologic brain microenvironment *in vitro* (57–61). The first report, published by Muffat and coworkers shows differentiation of both human embryonic stem cells and hiPSCs into early YS myeloid-like cells and subsequently into mature microglia-like cells within 74 days (60). Douvaras and coworkers published a protocol that induces myeloid differentiation of hiPSCs into CD14^+^/CX3CR1^+^ microglial progenitors which mature under IL-34 and granulocyte macrophage colony-stimulating factor (GM-CSF) conditions into microglia in 60 days (58). Two other protocols describe the induction of a two-step differentiation process from hiPSCs into hematopoietic progenitor-like CD34/CD43/CD45^−^ positive cells into microglia-like cells (57, 61). Haenseler and coworkers developed a protocol which induces the differentiation of hiPSCs into embryoid bodies followed by embryonic macrophage precursors in an M-CSF-, IL-3*-*dependent manner. Co-culturing of these hiPSC-derived macrophage precursors and cortical neurons in the presence of IL-34 and GM-CSF yields microglia-like cells within 14 days (59).

Human iPSC-derived microglia-like cells show several features of microglia *in vivo*: an amoeboid as well as a ramified microglia morphology, expression of microglial markers *IBA1, CX3CR1, CD11b, CD45*, and typical microglial functions such as phagocytosis, process motility, secretion of cytokines in response to LPS, IL-1β, or INF-γ stimulation, release of intracellular Ca^2+^ in response to adenosine diphosphate (ADP) and cell migration toward ADP and into 3D brain organoids (57–61).

To summarize, currently several protocols exist to obtain iPSC-derived microglial cells that partially approximate *in vivo* microglia.

### The Transcriptome of *In Vitro* and *Ex Vivo* Microglia Is Different

Although hiPSC-derived microglia display specific *in vivo* microglial functions and express a proportion of the homeostatic microglial gene signature (57–61), gene expression profiles of hiPSC-derived microglia-like cells do not fully match those of *ex vivo* human fetal and adult microglia. Principal component analysis (57, 59, 60) and hierarchical clustering (58) of gene expression profiles revealed that hiPSC-derived microglia-like cells are most similar to cultured primary human microglia (57–60), and much less resemble non-cultured *ex vivo* human fetal microglia (61). This finding is confirmed when the transcriptome of murine embryonic stem cell-derived microglia is compared to the transcriptomes of *ex vivo* and *in vitro* murine microglia (62). Thus, the *in vitro* environment strongly determines their gene expression profile, leading to a microglial phenotype different from *ex vivo* microglia.

More specific analysis of *in vitro* cultured murine and human microglia revealed a significant upregulation of inflammatory and stress-related genes (30, 43, 63). Cultured microglia show repression of homeostatic microglial signature genes (2, 30, 43, 63, 64) and genes associated with microglial development (43, 63) already after 6 h in culture (43). In addition, *in vitro* conditions cause a reduced expression of microglial genes associated with different neurodegenerative diseases, e.g., *TREM2*, in human microglia (43). The *in vitro*-induced transcriptomic changes are mirrored by the observation of a remodeled epigenetic landscape of *in vitro* microglia. Microglia that were cultured for 7 days lost more than 50% of the super-enhancers identified in *ex vivo* microglia, including the *SALL1* super-enhancer (43), that regulates microglia identity and function (65). A decrease in H3K27Ac levels (associated with active transcription) is observed at accessible (ATAC-enriched) regions for microglial TFs, indicating an *in vitro*-induced loss of microglial (super-) enhancer activity. This finding is corresponding with the downregulation of *ex vivo* microglia-specific genes and TFs (43).

These findings suggest that cultured microglia are less mature and have a more inflammatory phenotype than *ex vivo* human microglia. As a consequence, transcriptomic discrepancies between *ex vivo* and *in vitro* microglia are challenging the validity of current *in vitro* microglia culture systems to study murine and human microglia physiology and pathology.

Interestingly, Spaethling and coworkers were able to distinguish different brain cell types (neurons, oligodendrocytes, astrocytes, and microglia) after surgically obtained brain tissue was kept in culture for 3 weeks, with k-means clustering of cell-specific markers based on single-cell RNA sequencing data. Although the comparison between cultured microglia with *ex vivo* microglia has not been performed, the current results suggest that *in vitro* microglia lose many genes belonging to the homeostatic gene signature, though microglia can still be segregated from other brain cells in single-cell sequencing analysis (66).

Various studies have aimed to reveal crucial microenvironmental factors in *in vitro* culturing systems, that drive microglial identity toward a more *in vivo*-like state (30, 63). Whereas the astrocyte-secreted factors Tgf-β2, Csf1, and cholesterol are identified as microglial survival factors *in vitro* (63), Tgf-β supplementation of culturing media only led to partial (2, 30, 43) or no (63) re-establishment of an *ex vivo*-like microglial gene expression profile in cultured microglia.

Concluding, the gap in conformity between gene expression profiles of *ex vivo* and *in vitro* microglia is likely caused by the lack of yet unknown CNS microenvironmental factors that keep microglia in a homeostatic state in currently used *in vitro* culture systems.

### Microglia Isolation Procedures Affect Their Transcriptome

Next to the *in vitro* cell culture conditions, it should be of note that other *in vitro* procedures, such as the isolation procedure of microglia (tissue dissociation and cell sorting) can affect their gene expression profile, leading to the possibility that currently used reference *ex vivo* microglia expression data might not completely reflect *in vivo* microglia. In fact, it was shown that microglia inflammatory activation markers (*Il1b, Tnf*, and *Ccl2*) are already upregulated prior to placing murine microglia into culture, indicating that the isolation procedure by itself already affects the microglial phenotype (63). In addition, evidence indicates that enzymatic dissociation of brain tissue at 37°C leads to the upregulation of inflammatory genes in microglia when compared to mechanical dissociation at 4°C [(29); Eggen et al., unpublished data] or to the cTag-PAPERCLIP method (based on genetically modified animals and does not require tissue dissociation) (67).

In order to minimize the introduction of *in vitro*-induced artifacts when working with microglia in culture, the development of culture conditions and dissociation procedures that result in *in vitro* microglia that highly resemble microglia *in vivo* is of great importance.

## Microglial Activation States from a Transcriptome Point of View

### Functional and Morphological Aspects of Microglial Activation

“Microglial activation” is an umbrella term commonly used to describe a great variety of functional and morphological responses of microglia toward different triggers including stress (= homeostatic imbalance), inflammation, or chronic neurodegenerative conditions.

While this term implies that microglia are in a dormant state under healthy/homeostatic conditions, already more than 10 years ago, two photon-imaging of the mouse cortex *in vivo* showed that microglial protrusions are highly motile in order to scan their microenvironment for harmful exogenous and endogenous danger signals (68, 69). In addition, microglia are highly motile under healthy conditions during development. Synaptic pruning, the elimination of excessive, non-active neurons formed early in development, is realized by complement-dependent phagocytic activity of microglia (20, 21).

Microglia in a healthy brain are characterized by a small soma from which ramified protrusions are extending a morphology evolutionary conserved in different species (70). Classically, microglial activation was associated with an amoeboid-like morphology that enables microglia motility and phagocytic function (22). However, morphological transformation of microglia upon a shift in activation state does not seem to be uniform. Microglial morphologies range from amoeboid-like under inflammatory conditions (71) to hyper-ramification in response to stress (72) and accelerated aging (73), with many intermediate morphologies in between. In addition, different microglia morphologies can also be present at a defined condition such as stroke (74). Thus, it seems that there is, based on morphology, a yet unclear number of microglial activation states and single-cell resolution experiments are required to address that issue in detail. Since the discovery of microglia a century ago, we are aware of the wide range of morphologies microglia can adopt (12), though for most conditions direct links between a specific morphology and functionality of microglia are still unknown.

### A Brief History of Categorizing Concepts for Macrophage and Microglial Activation

An early concept that was first postulated for peripheral macrophages is the dichotomous categorization of macrophage activation states into classical activation (M1) or alternative activation (M2), analogous to the Th1 and Th2 nomenclature of T-lymphocytes (75). In an attempt to structure the complexity of microglial activation, the same M1-M2 classification was applied to activation states of microglia. The M1 phenotype is characterized by the production of pro-inflammatory cytokines (*Tnf-α, Il-6*, and *Il-1β*), chemokines, and reactive oxygen species leading to an acute immune response. The M2 phenotype is characterized by the production of anti-inflammatory cytokines (*Il-4* and *Il-13*) and facilitates debris clearance, wound healing, and restoration of brain tissue homeostasis (76, 77). It was assumed that microglia react to a stimulus with an M1 phenotype to address pathology and damage, followed by transition to an M2 phenotype in order to execute tissue repair (78). More detailed understanding led to the acceptance that microglial activation states are diverse and that intermediates between M1 and M2 phenotype exist (76). Further development of this concept in the macrophage field suggested to refine the M1-M2 nomenclature by adding the triggering stimulus as an abbreviation to the M1 or M2 classification (77).

Transcriptome studies revisited this concept by disproving the existence of the mutual exclusive M1-M2 polarization states. M1-M2-associated genes (77) were co-expressed by murine monocyte-derived brain macrophages/microglia in the context of traumatic brain injury (79) and ALS (31). Moreover, transcriptome-based network analysis of human monocyte-derived macrophages exposed to 29 different stimuli *in vitro* revealed that each stimulus triggered the expression of a distinct transcription profile. These profiles expand far beyond the M1-M2-associated transcription profiles and under some conditions, M1- and M2-markers are solely expressed at baseline level (80). This study indicates that the concept of an activation spectrum in between the M1-M2 extremes is inadequate.

Recent studies have thus led to the abandonment of this static and outdated M1-M2 concept of microglial (81) and macrophage activation (82) states and point toward the adaption of a so-called “multidimensional concept.” This concept incorporates ontogeny, microenvironmental signals, as well as present and past endogenous and exogenous stress signals (83). Such a concept would be in line with current knowledge gained from (single cell) transcriptome and epigenome studies about the great variety of microglial activation states specific to different conditions including aging and neurodegenerative diseases (84).

### The Microglial Transcriptome During Aging

#### Murine Aged Microglia

During aging, microglia undergo several phenotypic changes including in morphology and function (85). The microglial phenotype in aging was extensively studied in a mouse model of accelerated aging that is marked by genotoxic stress due to deficiency of the DNA-repair protein Ercc1. Microglia in generic *Ercc1* mutant mice have a hyper-ramified morphology accompanied by increased proliferation rates. Upon LPS stimulation, microglia from *Ercc1* mutant mice show enhanced expression of pro-inflammatory cytokines (*Il-1β, Il-6*, and *Tnf-α*), enhanced phagocytic activity, and reactive oxygen species production when compared to wild-type mice. This exaggerated responsiveness of microglia in aged and in *Ercc1* deficient, accelerated-aging mice is referred to as priming. The primed immune state was confirmed by transcriptional profile analysis, identifying an upregulation of genes associated with immune-related signaling pathways (73). This microglial phenotype was also observed in mice where the *Ercc1* deficiency was targeted to forebrain neurons. These data suggest that genotoxic stress in neurons could induce the observed primed state in microglia.

Overall, aging seems to induce a phagocytic and antigen-presentation gene expression profile when microglial transcriptomes of young and old mice are compared. Microarray analysis of pure microglia from young and old mice showed that aged microglia obtain a gene expression profile that is characterized by upregulation of genes involved in phagocytosis (including *Clec7a* and *Axl*), antigen processing and antigen presentation, interferon and cytokine signaling, as well as lipid homeostasis (including *Apoe*). The increased phagocytic activity in aged/senescent microglia is confirmed by a functional phagocytosis assay. Primed microglia are primarily detected in the white matter of the aging murine brain (86).

Similarly Orre and coworkers (87), identified 482 genes (e.g., *Slp1, Apoe, Il1r2*, and *Ccr6*) more abundantly expressed in cortical microglia at least by twofold in aged mice (15–18 months) when compared to younger mice (2.5 months). These genes are involved in processes such as vesicle release, zinc ion binding, positive regulation of cell proliferation, lymphocyte activation, and inflammatory response, indicating increased microglia-neuron signaling and an inflammatory status within the aging murine brain (87).

Interestingly, microglia in different regions of the mouse brain show divergent sensitivities to aging. Mainly genes involved in immune regulatory processes are differentially expressed in microglia upon aging. Cerebellar microglia seem to be most prone to aging-induced transcriptional differences, as they differentially express more than the double number of genes at 22 months of age, when compared to cortical, hippocampal, and striatal microglia of that age. Most of the differentially expressed genes in 22 months old microglia were upregulated genes involved in immunoregulatory functions. Age-related transcriptomic changes in cortical and cerebellar microglia occur relatively consistent during early (4–12 months) and late (12–22 months) aging. Gene expression changes during early aging (4–12 months) are most prominent in the striatum and during late aging (12–22 months) in the hippocampus. Microglia lose the expression of homeostatic microglial signature genes such as *P2ry12/13, Tmem119*, and *Fcrls*, most prominently in the cerebellum and to a lesser extent in the hippocampus, cortex, and striatum. These findings suggest that in addition to age-induced effects on microglia in the white matter, age-associated changes in microglia occur in a brain region-specific manner (40, 86).

In contrast to the general notion that microglia obtain a primed profile (40, 86, 87) and are neurotoxic during aging and age-related diseases (88), Hickman and coworkers identified a neuroprotective gene expression profile of microglia derived from the entire brain in aged mice (24 months) due to upregulation of genes involved among others in the Stat3 and Neuregulin-1 pathways. Aging affects the microglia sensome: receptors for endogenous ligands are downregulated while receptors for microbial ligands are upregulated (33).

#### Human Aged Microglia

An age-related increase in immunoreactivity for inflammatory-related microglial markers, CD68 and HLA-DR, as well as increased binding of a PET tracer for activated microglia ([^11^C]-(R)-PK11195) has been identified in the white matter of human postmortem brain tissue (86). Whereas these findings indicate that similar to mouse, human microglia adopt a more activated phenotype during aging, transcriptomic analysis identified that the overlap in genes that change expression during aging in mouse and human is very limited. Of note, mouse and human microglia overlap extensively with respect to the expression of homeostatic signature genes (39).

In the human transcriptomic dataset of Galatro and coworkers (39), 572 genes are differentially expressed in relation to the age of the donor. 212 genes are increased and 360 genes decreased in expression, and many of these genes are related to cytoskeleton, motility, and immune response processes. The top 100 most differential expressed genes in human microglia during aging are associated with actin (dis)assembly, cell surface receptors, and genes involved in cell adhesion and axonal guidance. Upregulated genes are mainly associated with actin (dis)assembly and motility, indicating a loss of microglia motility and migration in aged human microglia, a factor that might contribute to age-related CNS diseases. Genes involved in cell adhesion and axonal guidance and the sensome cell surface receptors are partially upregulated and downregulated (e.g., *P2RY12*) (39).

The overlap in genes that are differentially expressed during aging between humans and mice is very limited (39). Only 14 upregulated genes overlapped between the human and mouse data and are involved in positive regulation of cell-matrix adhesion. Nine genes have a reduced expression during aging in both human and mice, identifying genes as *ETS1, SEMA7A, MRC2, PSTPIP1*, and *EMP2* (39). Concluding, the response of microglia to aging is different in mouse and human.

Although not yet completely understood, microglia seem to obtain an age-induced immune-activated phenotype during aging, which likely contributes to the pathology of neurodegenerative diseases including AD and PD (85, 88–90). In contrast to mice, human microglia also adapted their cytoskeleton signaling during aging (7).

### The Transcriptomic Point of View on Activated Microglial Phenotypes in Neurodegenerative Diseases

A shared feature among different neurodegenerative disorders is microglia-mediated neuroinflammation (91). This type of microglial activation is a first line of defense in the CNS, but is also described as harmful (91, 92). Microglial activation can be observed in different neurodegenerative diseases in which microglia obtain specific phenotypes.

#### Alzheimer’s Disease

Several AD studies reported activated microglia surrounding Aβ plaques (34, 35, 93, 94). Plaque-associated microglia in the 5XFAD AD-mouse model (co-expresses five mutations associated with familial AD) contain upregulated sets of genes that overlap with the primed microglia transcriptional profile (95), that is characterized by enrichment of genes involved in among others immune and phagocytic processes, like *Apoe, Axl*, and *Clec7a*. Key protein regulators of those upregulated genes are *Tyrobp* (*Dap12*) and *CD11c* (*Itgax*). Of note, plaque-associated microglia in 5XFAD mice show an upregulation of phagocytosis-associated genes. Interestingly, the same phagocytic markers, *APOE, AXL, TREM2*, and *HLA-DR* are shown to be higher expressed in microglia surrounding dense-core plaques of early onset AD human postmortem tissue, when compared to late onset AD (LOAD) (94). In contrast to the finding that the expression of *TYROBP* is unaltered between plaque and non-plaque-associated microglia of LOAD postmortem brain tissue (94), *TYROBP* is identified as a key regulator of microglial-associated genes, based on the construction of a molecular network from autopsied whole brain samples of 1647 LOAD and non-demented subjects (96). Kamphuis and coworkers identified two distinct subsets (CD11c^−^ and CD11c^+^) in the CD11b^+^ microglia population surrounding Aβ plaques in APP/PS1 mice. Transcriptional alterations are more abundant in the CD11c^+^ population when compared to CD11c^−^ microglia, including an upregulation of *Clec7a, Itgam, Ctsb*, and *Cst7* expression. The CD11c^+^ microglial population is enriched for genes involved in a dampened immune response, carbohydrate and lipid metabolism, phagocytosis and lysosomal degradation, suggesting that the CD11c^+^ population is active in the clearance of amyloid deposition by possibly increased phagocytic and lysosomal activity and restriction of the inflammatory response (93). By contrast, it was recently observed that innate immune activity (inflammasome activity) of microglia leads to Aβ accumulation in APP/PS1 mice. Although not distinguishing between CD11c microglia subsets, it is shown that microglia secrete inflammasome-associated adaptor proteins, called apoptosis-associated speck-like proteins containing a CARD (caspase recruitment domain; ASC). ASC proteins can go through a cascade of modifications that lead to the assembly of large extracellular paranuclear ASC protein complexes, called ASC specks. These ASC specks are prone to bind Aβ deposits throughout brain tissue of AD patients and APP/PS1 transgenic mice. They are identified as the key contributors to several AD characteristics, such as the formation of plaques and spatial memory loss (97).

Concluding, the contradiction between the hypothesized function of CD11c^+^ microglia (clearance of Aβ plaques) and the proven function of CD11b^+^ microglia (augmentation of Aβ plaques), might be explained by the fact that CD11c^+^ microglia only constitute approximately 23% of the total activated CD11b microglial population (93), whereby its potential neuroprotective function might be overruled by the neurotoxic function of the remaining microglia.

Interestingly, single-cell analysis of hippocampal microglia from CK-p25 mice, a mouse model of severe AD-like neurodegeneration, identified a stepwise microglial gene expression trajectory in response to neurodegeneration. One week after CK-p25 induction, microglia possess an early-response state, which is hallmarked by an upregulation of genes involved in cell cycle, DNA replication, and repair. Increased incorporation of the thymidine analog EdU (5-Ethynyl-2′-deoxyuridine) and microglial density in CK-p25 mice 1 week after induction confirmed microglial proliferation in response to early neurodegeneration. Two and six weeks after disease induction, late-response microglia, show upregulation of immune response-related genes, such as *Ccl3/4, Apoe, Axl*, and *H2D1*. This microglial phenotype can be divided into two immune-activated subtypes that are marked by co-regulated genes induced by interferon type I (antiviral and interferon response genes) and II (MHC-II complex-related genes), respectively. Whether these microglial phenotypes have neuroprotective or neurotoxic functions remains unknown (98).

#### Multiple Sclerosis

Multiple sclerosis lesions have been categorized into (1) pre-active lesions, characterized by microglia activation in the absence of overt demyelination, (2) active lesions with massive infiltration of microglia and monocyte-derived macrophages, (3) mixed active/inactive lesions that consists of a hypocellular center and a foamy macrophage/microglia-enriched rim with partial demyelination, and (4) inactive lesions that are absent of cells and completely demyelinated. MS lesions are surrounded by normal appearing white matter, where microglial activation may occur as well (99). It is very difficult to interpret the distinct roles of microglia and monocyte-derived macrophages in MS pathology, since both macrophage populations are present in MS lesions (6). This mixed cell population of microglia and monocyte-derived macrophages has been investigated on transcriptomic level in human MS tissue (38, 100). Attempts have been made to decipher the role of microglia and peripheral monocytes in an experimental autoimmune encephalomyelitis (EAE, myelin-oligodendrocyte-glycoprotein peptide (MOG)-induced) mouse model for MS (37, 101). Yamasaki and coworkers distinguished microglia from monocyte-derived macrophages by the use of genetically modified mice that express fluorescent proteins (green or red fluorescent proteins) expressed under the control of a microglial (Cx3cr1) or monocytic (Ccr2) promoter. The study showed that microglia and monocyte-derived macrophages from the same tissue have different phenotypes in EAE. At the onset and peak of disease, microglia upregulated genes that are involved in the complement system (e.g., *C1qa, C3*, and *C4*), chemotaxis (e.g., *Ccl2*/*4*), cell migration, and acute inflammation (e.g., *Il1β* and *Tnf*) and downregulated genes that are involved in cell metabolism. By contrast, the gene expression profile of monocyte-derived macrophages is characterized by phagocytosis-, autophagy-, and cell clearance-related genes. Along with the finding that solely monocyte-derived macrophages form contacts at nodes of Ranvier, it was suggested that monocyte-derived macrophages initiate demyelination at EAE onset, whereas microglia seem to be responsible for the attraction of monocyte-derived macrophages to the CNS and to clear debris (37). Similar results have been reported in a study that used CD44 protein expression levels to distinguish microglia (CD44^low^) from monocyte-derived macrophages (CD44^high^) in EAE. RNA expression analysis of microglia and monocyte-derived macrophages from EAE mouse brain tissue reveal that macrophages display a more pronounced immune activation phenotype at the peak of EAE, characterized by common activation markers such as *MHCII, CD40*, and *CD86*. In comparison with monocyte-derived macrophages, microglia upregulate genes involved in uptake of apoptotic cells, the complement signaling, and chemotaxis at the peak of EAE (101).

In conclusion, these above studies suggest that monocyte-derived macrophages and microglia have different roles during the disease progression of EAE. Monocyte-derived macrophages seem to be the mediators of demyelination, whereas microglia are primarily responsible for the induction of peripheral infiltration to the CNS and clearance of apoptotic neurons in EAE. Whether these different macrophage phenotypes exist in human MS pathology as well needs to be addressed in future experiments.

#### Parkinson’s Disease

Microglial activation is initiated by several components, whereas one of the most frequently altered genes in familial PD is α-synuclein (91). Overexpression of a-synuclein led to the activation of BV2 cells, a microglial cell line, measured by an increase in cytokine production (102). Since *in vitro* cultured microglia do not resemble *in vivo* microglia (see The Transcriptome of *In Vitro* and *Ex Vivo* Microglia Is Different), the microglial phenotype associated with PD *in vivo* yet remains to be elucidated.

Whole tissue lysate small RNA sequence analysis of postmortem prefrontal cortex of PD patients (demented and non-demented) and control subjects, identified a set of 29 PD-related miRNAs (103). Interestingly two *Pu.1* related micro RNA’s (miR146a and miR-155) (36, 104) are upregulated in PD subjects, suggesting that microglia might be activated in human PD. Single-cell laser captured microglia of human postmortem PD brain tissue were used to identify microglial gene expression in PD (45). Overall, the most differentially expressed genes in microglia derived from PD subjects compared to control subjects are involved in functions such as aldosterone synthesis and secretion, positive regulation of protein complex assembly, focal adhesion assembly, tonic smooth muscle contraction, and positive regulation of reactive oxygen species biosynthetic processes. 313 genes are differently expressed in microglia located in the substantia nigra when compared to the CA1 hippocampal region of PD patients. These genes are involved in the behavior, regulation of transport, and synaptic transmission processes. The above findings indicate regional differences in microglial functioning in PD (45). Concluding, overall the expression pattern of genes in PD points toward microglial activation. Unfortunately, the small number of PD microglia transcript studies limit the current conclusion.

#### Amyotrophic Lateral Sclerosis

An activated microglial phenotype was reported in the transgenic SOD1-G93A mouse model for ALS, that contains mutations in the human superoxide dismutase 1 gene. The microglial phenotype is identified as a neurodegeneration-specific phenotype and differs from LPS-activated microglia as well as from M1- or M2 macrophages (31). It was shown that microglia in SOD1-G93A mice simultaneously upregulate neurotoxic and neuroprotective factors as well as pro-inflammatory-related genes (e.g., *Tnfα* and *Il-1β*). In addition, an upregulation of genes that have been associated with AD, including *Trem2, Tyrobp*, and *Apoe* associated with AD are found in microglia from SOD1-G93A mice. Furthermore, *Apoe* is also upregulated in both SOD1-G93A mice and ALS subjects (sporadic and familial) (36). At least for *in vitro* conditions, *Apoe* seems to play a role in forcing the “surveilling” microglia toward an immune-activated (M1-like) phenotype (36). In addition, a downregulation of the homeostatic microglial signature genes (including *P2ry12* and *CD39*), transcriptional factors (including *Egr1, Atf3, Fos*, and *Mafb*), developmental genes (such as *Tgfb1, Tgfbr1*, and *Csf1r*), and genes related to phagocytic ability and cell migration was described in SOD1-G93A mice (36). This indicates a suppression of several homeostatic microglial functions. Interestingly, the microRNA-155 (miR-155) is identified to be upregulated in microglia of SOD1-G93A mice as well as in spinal cord tissue of ALS subjects (36).

Genetic ablation of miR-155 in SOD1 mice causes a delay of the disease onset, an extend of the animal survival rates, and reversed the expression of SOD1-related upregulation of inflammatory genes and downregulation of homeostatic genes in microglia. In conclusion, miR-155 seems to be an important factor in driving the phenotypic switch from homeostatic to SOD1-specific activation microglia. Therefore, miR-155 might be a potential new therapeutic target in ALS (36).

## Microglial Core Gene Signatures Associated with Different Diseases

Next to the identification of disease-specific microglial transcriptomes, in the past years several studies have addressed a core profile of microglial genes that are dysregulated in multiple neurodegenerative diseases (34, 35, 95).

Massively parallel single-cell RNA-sequencing of CD45^+^ immune cells revealed among others the presence of three novel microglial transcriptional subpopulations in 5XFAD mice that are not present in wild-type animals. Two of them are characterized by the expression of genes involved in lipid metabolism and phagocytosis and are specifically located near Aβ plaques in the cortex of AD mice, called disease-associated microglia (DAM). DAMs seem to undergo a cascade of subsequent changes in gene expression profiles alongside the progression of the disease. The first step includes an increased expression of *Tyrobp, Apoe*, and *B2m* and reduced expression of microglial homeostatic signature genes (*Cx3cr1* and *P2ry12*). There seems to be a *Trem2*-dependency from the second step onward together with the upregulation of genes involved in lipid metabolism and phagocytic activity, such as *Lpl, Cst7, Axl*, and *Clec7a*. DAMs have also been identified in postmortem human AD tissue and in a mouse model for ALS (mSOD1 mice). Moreover, appearance of DAMs was observed when CD11b^+^ immune cells are compared between brains of young (7 weeks old) and aged (20 months old) mice. These findings suggest that DAMs (35) might have a general neuroprotective function involved in the clearance of accumulating proteins observed in aged and age-related neurodegenerative diseases brain tissue (105).

In contrast to the hypothesized neuroprotective function of DAMs, investigation of bulk microglial transcriptomes in different disease models led to the identification of a microglial neurodegenerative/-toxic (MGnD) phenotype that is dependent on the Trem2-Apoe pathway. These MGnD are found adjacent to Aβ plaques in APP/PS1 mice and human AD postmortem brain tissue and in SOD1, EAE, and aged (17 months) mice. Two major transcriptional changes are observed in MGnD: (1) the downregulation of microglial homeostatic genes [including *Tgfb(r), Hexb, P2ry12*, and *Cx3cr1*] and TFs (including *Mef2a, Mafb*, and *Sall1*) and (2) the upregulation of inflammatory genes (including *Axl, Itgax, Clec7a*, and *Apoe*), leading to a switch from a homeostatic to a neurodegenerative/-toxic microglial phenotype. This switch seems to be induced by the phagocytosis of apoptotic neurons and is dependent on Trem2-Apoe signaling and accompanied by a suppression of the Tgfβ pathway. Depletion of *Trem2* in APP/PS1 and SOD1 mice suppresses the expression of inflammatory genes, including *Apoe*, restores the homeostatic microglia gene signature and functions, and also alleviates disease-specific characterizations such as reduced Aβ plaques in APP/PS1 mice and reduced expression of miR-155 in SOD1 mice. Interestingly, the microglial homeostatic phenotype seems to be preserved in human AD patients who carry a mutation in the TREM2 gene when compared to AD patients with wild-type TREM2 (34).

Compared to the results of Krasemann and Keren-Shaul, a similar set of genes, including *Apoe, Axl, Itgax, Lgals3, Clec7a, MHC-II*, and *Cxcr4*, was identified as a commonly upregulated network of genes in different mouse models of aging [physiological aging and accelerated aging (24 months; Ercc1 deficient)] and murine disease models (APP/PS1 and SOD1) when compared to acute immune activation with LPS. This network is classified as the “microglia priming” network and is associated with functions involved in AD signaling, antigen presentation, lysosome and phagosome pathway. In addition, it was found that microglial homeostatic genes are suppressed in this “primed” network. The “primed” gene expression network related to microglial activation is contrasting with an “acute” activation network, specific for acute microglial inflammatory response to LPS that is marked by an upregulation of genes involved in ribosome, TLR signaling, and NOD-like receptor signaling (95).

Summarizing, different studies identified a microglial gene signature associated with multiple diseases that is marked by the downregulation of microglial homeostatic signature genes and the upregulation of genes associated with inflammation, phagocytosis, and lipid metabolism, whereby the two genes *Apoe* and *Trem2* seemed to be crucial players. Whereas the upregulation of phagocytic genes might imply neuroprotective functions of microglia (35, 93), recent studies show that microglia also seem to have a neurodegenerative/-toxic function in multiple neurodegenerative diseases (34, 97).

**Figure 1:**
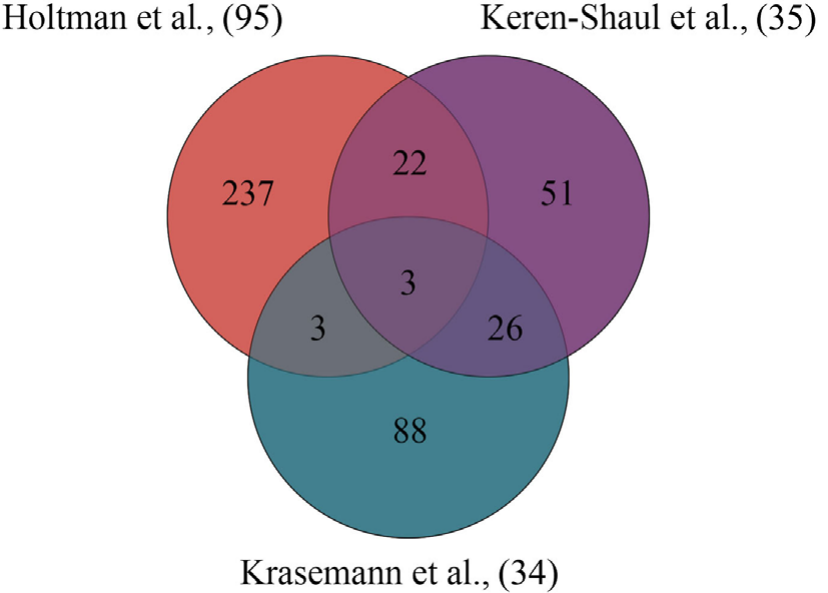
Overlapping gene signatures of microglial core profiles associated with multiple diseases identified in three independent studies. Using the “primed” module by Holtman and coworkers (95), the gene profile of disease-associated microglia identified by Keren-Shaul and coworkers (35) and the gene profile of the microglial neurodegenerative phenotype identified by Krasemann and coworkers (34), three genes were identified to be shared among the three microglia datasets associated with multiple diseases.

By comparing these aging- and neurodegeneration-associated microglial core gene signatures (34, 35, 95), an overlap between the different gene signatures (modules) is determined and visualized in Figure 1. A summary of the overlapping aging- and neurodegeneration-associated genes is depicted in the Venn diagram and are is listed in Table 1 where the annotation of gene functions is done with stringDB (106). There are three genes identified that are shared among these three datasets: *APOE, AXL*, and *IGF1*, identifying a very limited overlap of microglial-associated disease genes between three studies that have investigated similar age-related disease mouse models.

**Table 1:** Gene overlap of different microglial core profiles associated with multiple diseases.

Compared studies	Overlapping genes
Keren-Shaul et al. (35) and Krasemann et al. (34)	*BIN1, CCR5, CD34, CKB, CTSD, CX3CR1, ENTPD1, EPB4.1L2, F11R, FSCN1, GPR34, GPR56, LGMN, LTC4S, OLFML3, P2RY12, P2RY13, PMEPA1, RHOB, SERPINE2, SIGLECH, SLCO2B1, SPARC, SYNGR1, TMEM119*, and *TREM2*

Holtman et al. (95) and Keren-Shaul et al. (35)	*ANK, APLP2, B2M, CD52, CD68, CD9, CLEC7A, CSF1, CST7, CTSB, CTSZ, EEF1B2, GRN, GUSB, H2-K1, HIF1A, ITGAX, LGALS3BP, NPC2, PLD3, PSAT1*, and *TYROBP*

Holtman et al. (95) and Krasemann et al. (34)	*CXCL16, CCL5*, and *GAS7*

Holtman et al. (95), Krasemann et al. (34), and Keren-Shaul et al. (35)	*APOE, AXL*, and *IGF1*

## Discussion

The diversity of microglial phenotypes can be metaphorically imagined as the manifold compositions of colorful crystals seen in a kaleidoscope, whereby a change in (micro)environment is functionally equivalent to a rotation of the kaleidoscope. Technological developments in next-generation sequencing provide the possibility to reveal cell-type specific transcriptomes at single-cell resolution. Over the last 5 years, different studies have concordantly established the microglial gene signatures in mice and humans under homeostatic conditions (2, 3, 29–33, 39, 43). In addition, microglia-specific gene signatures in different neurological disease contexts were investigated. Summarizing, it was shown that the expression of many of the homeostatic microglial signature genes are repressed during aging and age-related CNS diseases. It is expected that more transcriptomic studies will follow, especially in regard to neuropsychiatric disease, from which transcriptome datasets are currently very sparse.

**Figure 2:**
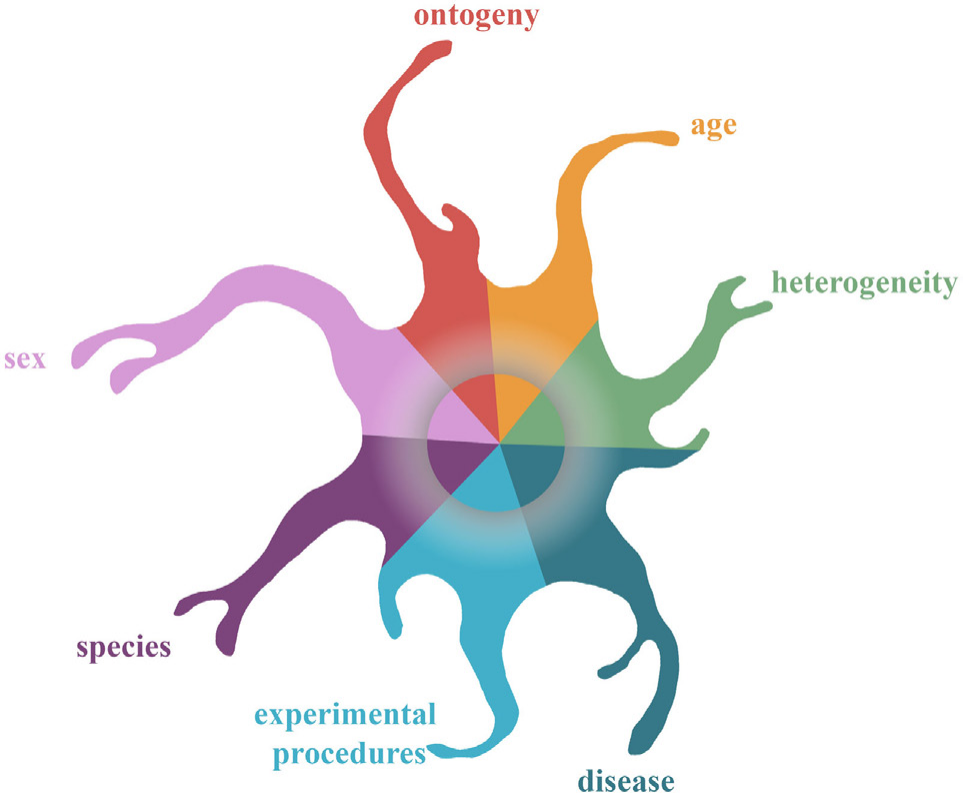
The kaleidoscope of microglial phenotypes: complexity of determining a microglial core profile associated with multiple diseases. This figure depicts examples of factors that impinge on the microglia epigenome and transcriptome. Seven factors are depicted that have been shown to influence the microglial transcriptome: brain region, ontogeny, disease, age, sex, experimental procedures, and species. The influence of these factors on the transcriptome converges in the center of the gray rim. This rim represents the imprint of these factors on the epigenome, which yet remains to be revealed.

Identification of microglial genes that are highly expressed and shared between different neurodegenerative diseases and aging might result in the identification of regulators that can alter affected pathways in multiple diseases. Thus far, three core signatures of microglia associated with multiple diseases have been identified (34, 35, 95). Although, microglial transcriptomes under similar conditions (aging and models for AD, MS, and ALS) have been evaluated in these three studies, only a limited overlap of genes is identified (see Figure 1), indicating that other factors might have influenced the transcriptional outcome. Figure 2 depicts examples of parameters, such as development, aging, sex, diseases, and experimental procedures, that were shown to influence microglial transcriptomes and might have led to the different results of the above-mentioned studies. In order to identify a “pure” microglial core signature related to multiple CNS diseases, the only deviating factor between the compared datasets should be “disease/disease model.” Obviously, this criterion is difficult to meet in a laboratory setup and is mere impossible in datasets derived from the human population. Conversely, the question arises whether the overlap of disease-associated microglial datasets, that are influenced by other factors, indeed identifies a universal regulator that is most important for all different investigated disease phenotypes. In view of the fact that research is often pursued in order to identify drug targets for human diseases, it is questionable if identification of a multiple disease-associated core signature of microglia will lead to a universal drug target for multiple diseases or whether disease-specific regulators might be more suitable as drug targets.

Current studies have delineated the influences of several environmental events (see Figure 2) on the microglial transcriptome. These findings indicated that microglia “imprint” different events on their (epi)genome and that imprints that occur early in life can possibly influence microglial phenotype over the entire lifespan of an animal. Although the precise microglial turnover rate is not yet completely clarified, microglia seem to be quite long-lived cells further supporting a potential role of epigenetics in regulating microglial function. Investigation of the microglial epigenetic landscape under homeostasis has already provided valuable insight in the (micro)environmental-induced dynamics of chromatin modifications and how these affect gene expression (2, 3, 43). Although currently unknown, investigation of the microglial epigenetic landscape in context of CNS diseases would reveal the link between the CNS-disease related microenvironment and the corresponding transcriptome and would definitely contribute to a better understanding of the role of microglia in the pathophysiology of CNS diseases.

One of the recent developments in sequencing technology is the ability to decipher transcriptomes and epigenomes at the resolution of a single cell (107, 108). The identification of single-cell transcriptomes and epigenomes will greatly facilitate the characterization of distinct microglial phenotypes under particular conditions, ranging from (ab)normal development to neurodegenerative disease, and allow for a detailed and sophisticated functional classification of microglial phenotypes.

## Author Contributions

MD and LK wrote the manuscript. BE and EB gave feedback and edited the manuscript.

## Conflict of Interest Statement

The authors declare that the research was conducted in the absence of any commercial or financial relationships that could be construed as a potential conflict of interest.
